# High-Density Linkage Maps from Japanese Rice *japonica* Recombinant Inbred Lines Using Genotyping by Random Amplicon Sequencing-Direct (GRAS-Di)

**DOI:** 10.3390/plants12040929

**Published:** 2023-02-17

**Authors:** Rym Fekih, Yohei Ishimaru, Satoshi Okada, Michihiro Maeda, Ryutaro Miyagi, Takahiro Obana, Kazuyo Suzuki, Minoru Inamori, Hiroyuki Enoki, Masanori Yamasaki

**Affiliations:** 1 Food Resources Education and Research Center, Graduate School of Agricultural Science, Kobe University, Kasai 675-2103, Japan; 2 Bioscience and Biotechnology Center, Nagoya University, Nagoya 464-8601, Japan; 3 Eurofins Genomics K.K., Tokyo 143-0003, Japan; 4 Toyota Motor Corporation, Toyota 471-8571, Japan; 5 Graduate School of Science and Technology, Niigata University, Niigata 950-2181, Japan

**Keywords:** Japanese rice, *japonica* subspecies, recombinant inbred lines, GRAS-Di genotyping, genetic linkage map

## Abstract

The genetic dissection of agronomically important traits in closely related Japanese rice cultivars is still in its infancy mainly because of the narrow genetic diversity within *japonica* rice cultivars. In an attempt to unveil potential polymorphism between closely related Japanese rice cultivars, we used a next-generation-sequencing-based genotyping method: genotyping by random amplicon sequencing-direct (GRAS-Di) to develop genetic linkage maps. In this study, four recombinant inbred line (RIL) populations and their parents were used. A final RIL number of 190 for RIL71, 96 for RIL98, 95 for RIL16, and 94 for RIL91 derived from crosses between a common leading Japanese rice cultivar Koshihikari and Yamadanishiki, Taichung 65, Fujisaka 5, and Futaba, respectively, and the parent plants were subjected to GRAS-Di library construction and sequencing. Approximately 438.7 Mbp, 440 Mbp, 403.1 Mbp, and 392 Mbp called bases covering 97.5%, 97.3%, 98.3%, and 96.1%, respectively, of the estimated rice genome sequence at average depth of 1× were generated. Analysis of genotypic data identified 1050, 1285, 1708, and 1704 markers for each of the above RIL populations, respectively. Markers generated by GRAS-Di were organized into linkage maps and compared with those generated by GoldenGate SNP assay of the same RIL populations; the average genetic distance between markers showed a clear decrease in the four RIL populations when we integrated markers of both linkage maps. Genetic studies using these markers successfully localized five QTLs associated with heading date on chromosomes 3, 6, and 7 and which previously were identified as *Hd1*, *Hd2*, *Hd6*, *Hd16*, and *Hd17*. Therefore, GRAS-Di technology provided a low cost and efficient genotyping to overcome the narrow genetic diversity in closely related Japanese rice cultivars and enabled us to generate a high density linkage map in this germplasm.

## 1. Introduction

Rice (*Oryza sativa* L.) is the staple food of more than three billion people [1], corresponding to more than half of the world’s population. Accordingly, it is considered as one of the most important crops in the world. In addition to its economic importance, rice has long served as a model system in monocotyledon, not only for research on plant development but also on cereal’s genomics, pathology, and physiology due to the fact of its sharing synteny with other cereals, such as wheat (*Triticum aestivum* L.), maize (*Zea mays* L.), and sorghum (*Sorghum bicolor*) [2,3,4]. Rice’s first drafts of genome sequences were published in 2002 for *japonica* cultivar Nipponbare [5] and *indica* cultivar 93-11 [6]. In 2005, a high-quality whole-genome sequence was published using a *japonica* cultivar Nipponbare (IRGSP 2005), offering to the scientific community one of the most accurate sequences available for crop species. In fact, as a result of the complete high quality genome sequencing of rice, its small genome size (estimated to be 398 MB), the availability of databases and tools for functional genomics, and the identification of new genes and quantitative traits loci (QTLs) of agronomical interest are becoming easier.

With the emergence of the next generation sequencing (NGS) technologies, researchers and scientists have been focusing on developing new and more efficient breeding strategies that combine high throughput phenotyping and genomic technologies to accelerate crop breeding [7,8,9,10]. Thusly, the isolation of new rice genes is becoming easier and more rapid, revolutionizing the world of genomics.

The spectacular advance in whole genome sequencing technologies revolutionized the way to detect genome-wide polymorphisms and allowed a large number of single-nucleotide polymorphisms (SNPs) to be identified from comparisons between genome sequences. Consequently, it became possible to genotype a large number of SNPs in ultra-high throughput, even among closely related temperate *japonica* cultivars [11].

Genotyping by random amplicon sequencing-direct (GRAS-Di) is a new genotyping technology for detecting SNP and amplicon markers using NGS technology [12].

In addition to its technical simplicity, GRAS-Di has the potential of generating a large number of polymorphisms, an important factor to be used as molecular markers for genetic analysis. This new technology, which has been recently successfully used to reveal genetic structure of mangrove fishes [13], also provided high reproducible results with a minimal loss of genotype data in various species, including wheat, soybean, tomato, potato, sugarcane, cow, chicken, tuna, and humans [14].

Genetic diversity is universally acknowledged as the foundation of each breeding effort. The advancement of crop improvement and the genetic analysis of complex traits have used segregating populations derived from crosses between distantly related cultivars. This approach allows the detection of quantitative trait loci (QTLs) and the isolation of the responsible genes [15]. During the study of rice genetics, a commonly used approach is to utilize a mutation identified in a subspecies *japonica* and cross it with an *indica* cultivar. The purpose of this approach is to identify a high number of genetic variations and subsequently use them to develop genetic markers. These markers can then be employed to test their association with the desired phenotype [16]. This approach addresses co-segregation of phenotypes and markers from the parents to progeny and is commonly known as “linkage study” [17].

Genetic linkage maps provide a linear order of molecular markers along all the chromosomes for a specific genome, and those are highly valuable in helping to study the co-segregation of phenotypes and markers from parents to progeny.

Many captivating genetic analyses that have used this conventional genetic mapping approach and generated segregating populations (F_2_ progeny or recombinant inbred lines (RILs)) are derived from the crosses between *japonica* and *indica* cultivars to localize QTLs and genes controlling important agronomic traits [18,19,20]. However, the genetic and molecular analysis of closely related rice cultivars within a subspecies, such as Japanese rice population, presents challenges. One major obstacle is the limited genetic diversity and low levels of DNA polymorphism present among cultivars. This narrow genetic diversity can impede the ability to identify useful genetic markers and make it difficult to isolate specific genes or mutations associated with a particular phenotype. Large studies on the population structure in Japanese rice population are conducted to reveal and to clarify the genetic relationship among Japanese rice cultivars. In this context, Yamasaki and Ideta [21] classified 114 Japanese paddy rice populations into six subgroups and provided useful sets of Japanese rice cultivars for genetic applications.

Koshihikari, an elite Japanese temperate *japonica* cultivar, is the most widely grown cultivar, accounting for 35% of the total cultivated paddy field in Japan [22,23]. It is characterized by an excellent eating quality, an early heading date, stronger cool temperature tolerance at the booting stage, and a stronger preharvest sprouting resistance compared with other *japonica* cultivars [24]. For all these features, the cultivation of Koshihikari cv. has expanded all over Japan for more than 35 years, and many Japanese temperate *japonica* cultivars are currently developed using Koshihikari as a donor parent [11].

Nested Association Mapping (NAM) population has been first designed for maize [25,26] and subsequently for other cereals, such as rice, wheat [27], and barley [28]. Japan is located within a wide range of latitude, extending from 20° to 45° north and of longitude extending from 122° to 153° east (Geography of Japan Wikipedia https://en.wikipedia.org/wiki/Geography_of_Japan, accessed on 15 February 2023). As a consequence, its environmental characteristics, such as day length, temperature, and humidity level, vary greatly among regions. Rice cultivars are, therefore, carefully chosen by breeders to adapt the local climate of each of the 47 prefectures of Japan.

To exploit the natural variation of diverse Japanese rice cultivars and landraces, our research group at the Food Resources Education and Research Center, Kobe University has been generating, over the past few years, a Japanese Rice Nested Association Mapping (JNAM) population composed of 3268 RILs, using the cultivar Koshihikari as a common parent. Four of these RIL populations were used in this study. Cultivar Fujisaka5 has an early mating allele and has a partial resistance to rice blast [29]; cv. Futaba, however, is known for its resistance to leaf blast but not panicle blast [30]. Cv. Yamadanishiki is a popular Japanese *sake* brewing rice cultivar [31], and Taichung 65 is known for its wide regional adaptability, its early heading date, and its potential high yield [32]. GRAS-Di was applied for genotyping of four RIL populations, RIL71, RIL98, RIL16, and RIL91 and their parents, Koshihikari/Yamadanishiki; Koshihikari/Taichung 65; Koshihikari/Fujisaka 5; and Koshihikari/Futaba, respectively, which have a narrow genetic diversity. The generated markers (SNPs and amplicons) of the four RILs were organized into linkage maps and compared with those generated by GoldenGate assay of the same RIL populations. The average genetic distance between markers showed a clear decrease in the four RILs populations when we integrated markers of both linkage maps. The successful localization of five QTLs for heading date using these genetic maps, demonstrated the efficiency of GRAS-Di technology in revealing hidden DNA polymorphism in closely related Japanese rice cultivars, confirming GRAS-Di as a valuable tool to enhance functional genomics and genetic breeding studies for species with narrow genetic diversity, such as Japanese rice cultivars and landraces.

## 2. Results

### 2.1. Analysis of GRAS-Di Sequencing

**Figure 1:**
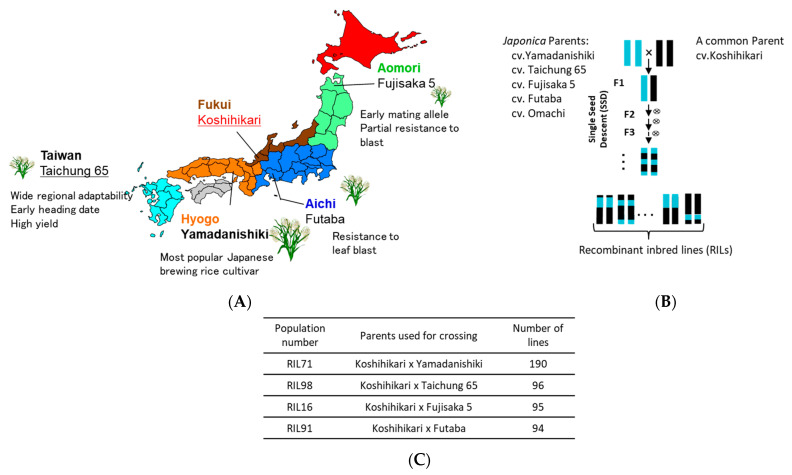
(**A**) Origin of Japanese rice parents used for generating Japanese recombinant inbred lines (RILs). (**B**) A simplified crossing scheme using single seed descend method (SSD) to generate the four RIL populations used in this study and number of their corresponding progeny lines (**C**).

Approximately 438.7 Mbp, 440 Mbp, 403.1 Mbp, and 392 Mbp called bases covering 97.5%, 97.3%, 98.3%, and 96.1% of the estimated rice genome sequence at average depth of 1× were generated after sequencing the recombinant inbred lines RIL71, RIL16, RIL91, and RIL98, respectively. The called base sizes of their corresponding parental lines, Koshihikari, Yamadanishiki, Fujisaka 5, Futaba, and Taichung 65 were 413.5 Mbp, 457.0 Mbp, 487.0 Mbp, 439.0 Mbp, and 373.0 Mbp, respectively (Table 1). The average percentage of Q30 bases (bases with a quality score of 30, indicating a 1% probability of an error and, thus, a confidence of 99%) was more than 92% for all reads from four RIL populations and their respective parents, and the average quality (GC: guanine–cytosine content) was at least 35.1% (Figure 1 and Table 1).

### 2.2. GRAS-Di Genotyping and Markers

Genotyping using GRAS-Di generated a total of 1050 (495 SNPs and 555 amplicons), 1285 (499 SNPs and 786 amplicons), 1708 (593 SNPs and 1115 amplicons), and 1704 (635 SNPs and 1069 amplicons) markers for RIL71, RIL98, RIL16, and RIL91, respectively (Supplementary Figure S2).

**Figure 2:**
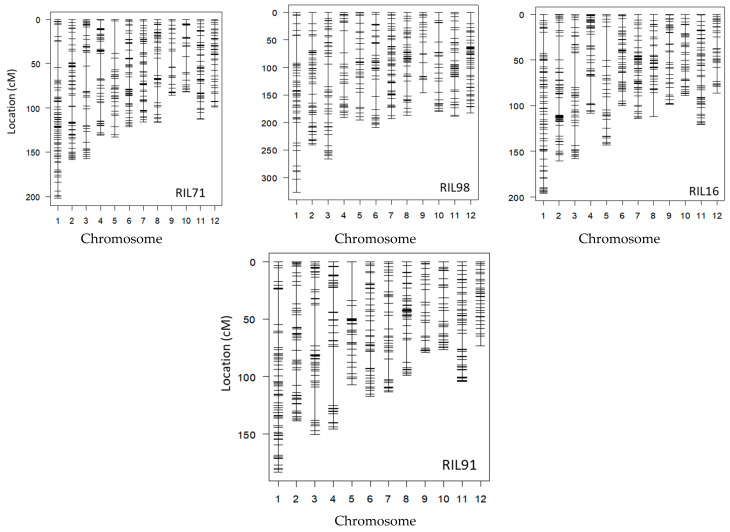
Linkage maps of the four RIL populations based on the integration of both markers generated by GoldenGate system and GRAS-Di technology, excluding the co-localized markers.

After the integration of all markers together, only one reliable marker was kept among a set of co-localized markers. Once the remaining co-localized markers were removed, we retained 527, 455, 501, and 436 markers for RIL71, RIL98, RIL16, and RIL91, respectively (Figure 2, Table 2). This suggested the location, on average, of one DNA marker every 700 kb, 820 kb, 744 kb, and 850 kb in RIL71, RIL98, RIL16, and RIL91, respectively (based on a genome size of Nipponbare reference sequence of approximately 373 Mb (IRGSP-1.0)). The integration map of all markers generated by both GoldenGate assay and GRAS-Di technology displayed a total of 1360, 1605, 2018, and 2056 markers for RIL71, RIL98, RIL16, and RIL91, respectively (Table 3).

The density of DNA markers, their distribution, and information on the integration linkage map for the four populations are summarized in Table 2. For RIL71, the total genome length is 1515 cM, including the above-mentioned 527 markers with unique map positions. The average distance between adjacent markers is 2.9 cM. Chromosome 7 is the most saturated, with an average distance of 2.3 cM. However, chromosome 5 is the least saturated, with an average distance of 4.7 cM. The longest chromosome is chromosome 1, with a total length of 202 cM, and the shortest is chromosome 10, with an average length of 81.7 cM. After excluding the co-localized markers, the integrated linkage map derived from genotyping of RIL98 population exhibits a total genome length of 1489 cM; the average distance between adjacent markers is 3.4 cM. Chromosome 1 is the longest, with an average length of 200.4 cM, and chromosome 9 the shortest one, with an average length of 90.4 cM. The most saturated chromosome is chromosome 7, with an average distance of 2.1 cM, whereas chromosome 10 is the less saturated, with an average distance 4.5 cM between markers. As for the integration map using RIL16 population, the full genome length is 1485.2 cM, and the average distance between adjacent markers is 3 cM. Chromosome 1 is the longest chromosome, with an average length of 195.7 cM, and chromosome 12 is the shortest one, with an average length of 86.3 cM. The most saturated chromosome is chromosome 6, with an average distance of 2.1 cM, and chromosome 3 is the least saturated, with an average distance 3.5 cM between markers. Integrated linkage map of RIL91 shows a full genome length of 1389.9 cM, with an average distance between adjacent markers of 3.4 cM. Chromosome 1 is the longest chromosome, with an average length of 183.6 cM, and chromosome 12 the shortest one, with an average length of 73.7 cM. The two densest chromosomes are chromosomes 11 and 12, with an average distances of 2.6 cM, whereas chromosome 4 contains the fewest markers, with an average distance of 4.7 between markers.

### 2.3. Genotyping by GoldenGate SNP Assay

**Figure 3:**
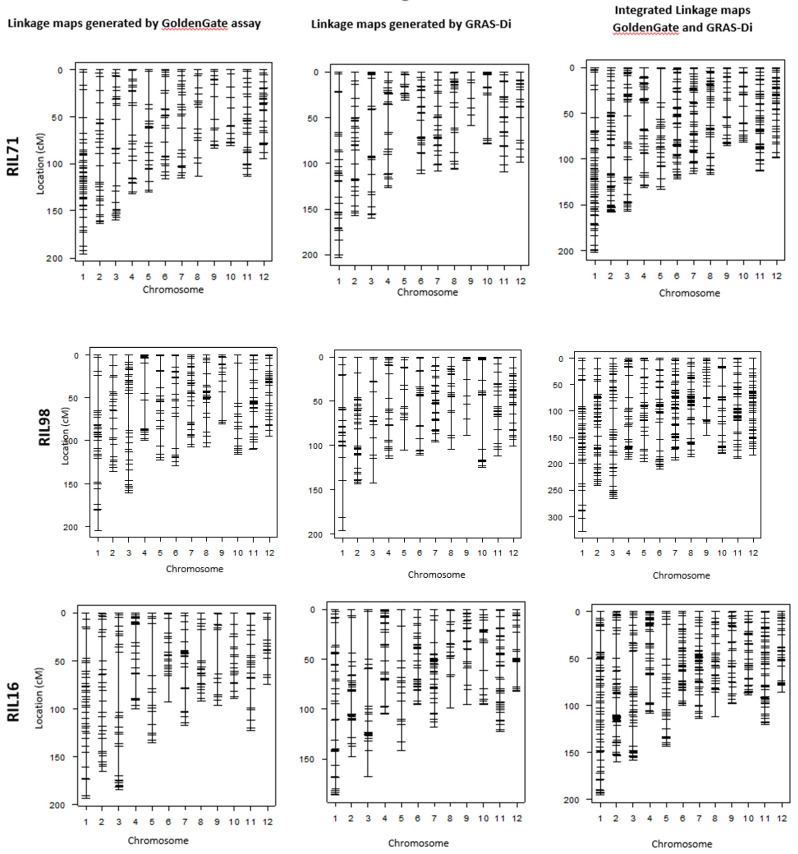

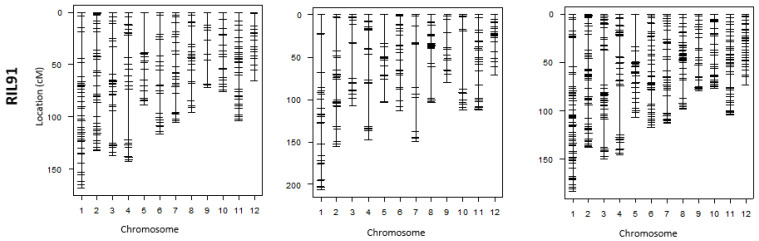
Individual linkage maps generated by GoldenGate and GRAS-Di and their corresponding integrated maps using the four RIL populations.

**Figure 4:**
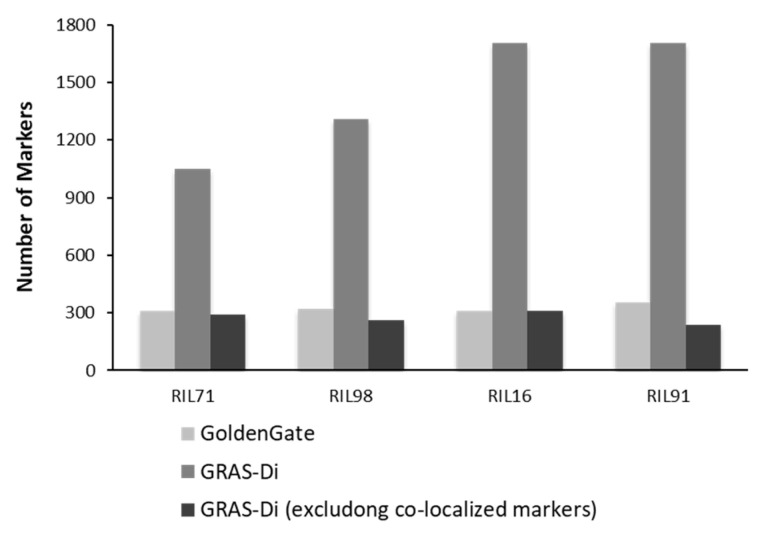
Comparison of number of markers generated by GoldenGate assay (including the co-localized markers) and GRAS-Di (including and excluding the co-localized markers).

In an attempt to confirm the increase of DNA marker information provided by GRAS-Di technology, we compared the linkage maps generated by GoldenGate assay with the integrated linkage maps generated by both GRAS-Di and GoldenGate assay (Figure 3 and Figure 4). The linkage map generated by GoldenGate assay provided a total of 292 markers in RIL71, 277 markers in RIL98, 262 markers in RIL16, and 286 markers in RIL91 (Table 4). The number of markers increased to 527 in RIL71 when we integrated makers generated by GRAS-Di and GoldenGate technologies together. Likewise, the integrated linkage maps from GRAS-Di and GoldenGate displayed a total of 455 markers in RIL98, 501 markers in RIL16, and 436 markers in RIL91 (Table 2).

Although there is a difference in markers density among chromosomes, the total number of DNA markers witnessed a clear increase. In each chromosome, the average distance between markers decreased from 5.3 cM to 2.9 cM in the integrated linkage map of RIL71, from 5.5 cM to 3.4 cM when we integrated all markers using RIL98, from 5.8 cM to 3 cM in the integration map of RIL16, and from 4.8 cM to 3.4 cM in the integrated map of RIL91. Moreover, markers generated by both GoldenGate SNP assay and GRAS-Di were checked for their correspondence in the four populations. A corresponding ratio of 99.8% was observed within the four RIL populations (Supplementary Figure S3), indicating that all linkage maps generated by GRAS-Di have a good agreement with those generated by GoldenGate assay.

Comparison of chromosomal sections lacking DNA markers (hereinafter referred to as “the largest gap”) in both maps (i.e., linkage map generated after genotyping by GoldenGate assay vs. linkage map genotyping by GRAS-Di) showed that the largest gaps became narrower after GRAS-Di genotyping. For instance, these largest gaps have been narrowed by up to 13.7 cM in the linkage map generated using RIL71 population (Table 3 and Table 4). The genotyping by GRAS-Di yielded new markers in seven chromosomes (1, 2, 3, 4, 8, 9, and 11) within the largest gap section of the individual linkage map generated by GoldenGate assay. Consequently, the size of these largest gaps was narrowed by 0.3–6.7 cM in the integrated map. In chromosomes 6, 7, and 12, new DNA markers were generated between markers having the largest gap. In chromosome 7, for instance, nine new markers were added to the previous individual linkage map (GoldenGate) resulting in smaller gap size in the integrated map. The same tendency was observed in the four other linkage maps. In the linkage map generated by GRAS-Di using RIL98 population, the largest gaps also become smaller in six chromosomes (chromosomes 1, 3, 4, 6, 9, and 10), and a maximum decrease of 24.2 cM was observed in chromosome 10 (Table 3 and Table 4). Moreover, the genotyping by GRAS-Di allowed for the identification of more DNA markers within the largest gap regions (Figure 3); for instance, the gap in chromosome 7 was filled with new markers, and the main gap was reduced from 19.4 cM to 9 cM (Table 3 and Table 4).

Likewise, the largest gaps in linkage maps of RIL16 and RIL91 were narrowed in many chromosomes, and new DNA markers filled the gaps generated by GoldenGate SNP genotyping. A reduction in largest gaps was noted in chromosomes 8, 10, 11, and 12 of the linkage map generated by GRAS-Di using RIL16 population and in chromosomes 5, 6, 9, and 10 of the linkage map generated by GRAS-Di in RIL91 (Figure 3). It is worth mentioning that the reduction of the “largest gap” reached 34.6 cM in chromosome 9 in the individual linkage map of RIL16 population. The maximum decrease in gaps reached 11.5 cM in chromosome 9 of RIL91. On the other hand, we observed a slight increase in the “largest gaps” of some chromosomes following the genotyping by GRAS-Di; the genetic distance of the largest gaps in chromosomes 5 and 10 of the linkage map of RIL71 population, for instance, increased from 34.9 to 36.6 cM and 17.9 to 18.0 cM, respectively. This increase also affected the largest gaps of other linkage maps, such as in chromosomes 2, 5, 8, and 11 of RIL98 population, in chromosomes 2 and 4 (chromosome 2: an increase from 25.5 to 36.7 cM, chromosome 4: an increase from 25.8 to 29.4 cM) of RIL16, and a slight increase in chromosomes 1, 3, 4, and 8 for the linkage map of RIL91 population (Figure 3; Table 3 and Table 4). Ultimately, following GRAD-Di genotyping, largest gaps of integrated linkage maps generated in this study have a genetic distance of approximately 30 cM (chromosomes 1, 3, 4, and 5 in the linkage map of RIL71; chromosome 1 in the linkage map of RIL98; chromosomes 2, 3, 4, 5, and 8 in the linkage map of RIL 16; and chromosomes 1, 3, 4, and 5 in the linkage map of RIL91). In addition, the largest gap in chromosome 12 in the integration map generated from RIL98 population as well as chromosomes 1, 3, 4, and 8 in the map of RIL91 slightly increased (Table 3). The corresponding genetic position, however, appeared different from the one in the map generated following genotyping by GoldenGate assay (Table 4). When we looked to the genotype data, it appeared that the recombination frequency between the newly acquired DNA markers (generated by GRAS-Di technology) and the adjacent DNA markers was higher than the recombination in the same region (largest gap) generated by GoldenGate assay, which explains the slight increase in the largest gap following GRAS-Di technology.

### 2.4. Identification of QTLs for Heading Date

**Figure 5:**
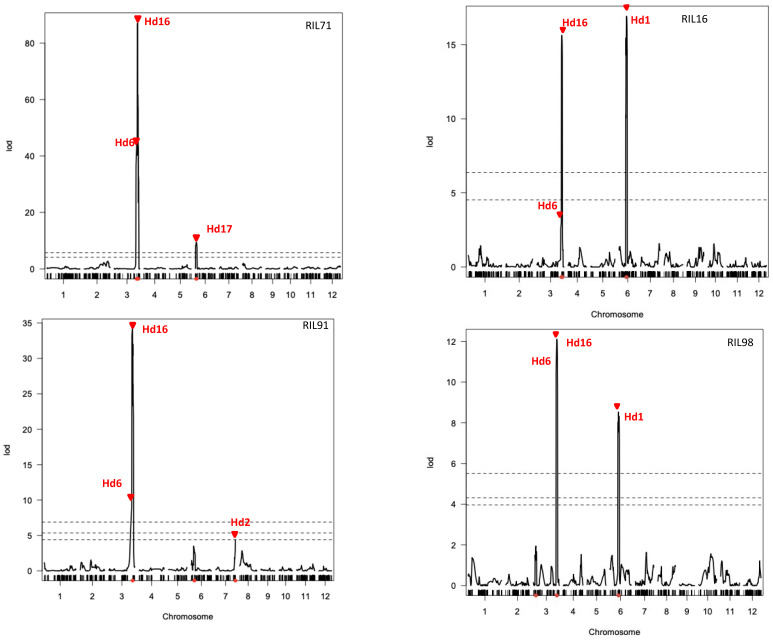
QTL analysis of heading date in four rice RIL populations using integrated linkage maps generated by GRAD-Di and GoldenGate. The dashed lines indicate 1%, 5% (RIL71 and RIL16), and 10% (RIL91 and RIL98) of genome-empirical thresholds.

Heading date (Hd) is an important trait for adaptation and expansion of rice to different cultivation areas. QTLs analysis for Hd was performed using the above linkage maps. As shown in Figure 5, significant LOD scores over the thresholds indicated the presence of QTLs in three chromosomes: chromosomes 3, 6, and 7. Peaks of LOD scores in these chromosomal regions were confirmed to be involved in heading date trait using the linkage map data of four RIL populations (Figure 5). The QTLs for *Hd6* and *Hd16* were detected on chromosome 3 using the four integrated linkage maps generated in this study. Additionally, three more QTLs were detected in chromosome 6 (*Hd1*, using linkage maps of RIL16 and RIL98; and *Hd17*, using linkage map of RIL71) and chromosome 7 (*Hd2*, using linkage map of RIL91).

## 3. Discussion

Most Japanese rice cultivars are of *japonica* origin. Previous studies classified Japanese rice accessions into *temperate* and *tropical japonica* using simple sequence repeats (SSR) markers [33]. Also using SSR markers, a previous study [21], provided an estimation regarding the genetic diversity of Japanese rice cultivars and demonstrated the presence of population structure, revealing the presence of six subgroups and admixture in the Japanese rice population. However, despite the phenotypic variation reported in Japanese cultivars [33], its genetic dissection remained limited because of the lack of molecular markers allowing the detection of polymorphism. The first molecular linkage map from *japonica* × *japonica* cross was developed by Redona and Mackill [34] using random amplified polymorphic DNA (RAPD) and restriction fragment length polymorphism (RFLP) markers and succeeded in the detection of QTLs for seedling vigor. To generate their *japonica* map, the authors used F_2_ progeny derived from the cross between cv. Italica Livorno and cv. Labelle. They suggested that regions in chromosomes 1 and 2 might lack polymorphism in *japonica* cultivars in contrast to chromosomes 10 and 11, which might be highly polymorphic among *temperate* and *tropical* group of *japonica* subspecies. Recent advances in genotyping technologies and associated reduced costs has changed the way to detect genome-wide polymorphism, since SNPs already replaced SSRs as the first DNA marker of choice [35]. These SNPs could be used to detect further DNA polymorphism among closely related cultivars; for instance, Yamamoto et al. [11] used whole genome sequencing of two closely related *japonica* rice cultivars, Koshihikari and Nipponbare, and collected over 67,000 SNPs between them. Subsequently, Nagasaki and collaborators [36] compared the genomic sequence of two other *japonica* cultivars (Eiko and Rikuu132) and Nipponbare as reference to construct a core set of 768 SNPs highly efficient and reliable for diversity and genetic analysis of biparental populations of Japanese rice accessions.

In the present study, Koshihikari cv. was used as a common parent to generate four RIL populations: Yamadanishiki is popular for its highest yielding and excellent brewing quality, which made it a good candidate as a crossing parent to conduct QTL analysis [31]. Taichung 65 is a *japonica* cultivar derived from the cross between Kameji and Shinriki; its potential for breeding includes early heading date (valuable trait for wide regional adaptability) and high yield [37]. Cultivars Fujisaka 5 and Futaba are, however, renamed mainly for their partial and higher resistance to leaf blast [29,30]. Over the generations, by crossing each of these four Japanese parent cultivars with Koshihikari cultivar, the final genotype of the generated RILs was a random shuffle of parental genotypes. Since each self-pollination reduces heterozygosity by half, the majority of genomes of RILs have become homozygous at advanced generations (F_6_–F_7_). Then, in order to reveal potential hidden polymorphism among Japanese rice cultivars, we used GRAS-Di technology to sequence and genotype these four RIL populations and their respective parents. The low cost and high accuracy of GRAS-Di allowed an accurate sequencing using 1× genome coverage of the four RIL populations and their respective parents (Table 1). GRAS-Di technology has randomly amplified multiple regions of the genome to generate amplicons that have been subjected to NGS sequencing [12]. Additionally, GRAS-Di genotyping of the F_6_-F_7_ of the four RIL populations and their respective parents confirmed that these populations were almost fixed to the homozygous state (data not shown) and generated a considerable number of markers used to display genetic linkage maps using data of four RIL populations. The total number of markers generated by GRAS-Di (amplicon and SNP markers) was compared with the number of SNP markers previously generated by GoldenGate assay. Although there was a difference in markers density between chromosomes, the total number of DNA markers witnessed a clear increase (Table 4). The distribution of DNA markers, however, is not uniform across the four linkage maps. Gaps between markers were localized in different chromosomes of the four linkage maps (Figure 2 and Figure 3). This uneven distribution of markers might be related to the low frequency of DNA polymorphism in these particular genomic regions, for instance, between Koshihikari and Yamadanishiki for individuals of RIL71 population, Koshihikari and Taichung 65 for individuals of RIL98 population, Koshihikari and Fujisaka5 for individuals of RIL16, and Koshihikari and Futaba for individuals of RIL91 population.

However, in this study, chromosomal regions displaying “SNP deserts” (term defined in [38]), such as in chromosome 5, and which have been related to rice domestication, have been relatively filled with markers in the integrated linkage map of RIL98 (Figure 3). The same region on chromosome 5 appeared empty of markers in the three remaining linkage maps derived from RIL71, RIL16, and RIL91. Moreover, a lower recombination rate around the centromere could also explain the reason of this distorted recombination and the low frequency of markers around these regions of the genome.

Overall, the average genetic distance between markers decreased in the four integrated linkage maps (Figure 3), but the total chromosome length did not significantly change when we compared displayed linkage maps using GoldenGate SNP markers and GRAS-Di markers. Consequently, the integrated maps provided higher density level of arranged markers. The corresponding ratio of 99.8% observed within the four RIL populations indicated that all linkage maps generated by GRAS-Di have a good agreement with those generated by GoldenGate assay. The comparison of largest gaps displayed in linkage maps generated by GoldenGate assay and GRAS-Di, showed a general narrowing of these gaps following the use of GRAS-Di, and this was due to generation of new markers within the largest gap regions. For instance, new markers were generated in seven chromosomes of the linkage map displayed when using RIL71 population, allowing a narrowing of up to 6.7 cM when we integrated both maps (Table 3 and Table 4). In the individual linkage map using RIL98, chromosome 7 witnessed a shrinkage of more than 10 cM following GRAS-Di genotyping. The reduction of the “largest gap” reached 34.6 cM in chromosome 9 in the individual linkage map of RIL16 population. Despite this general decrease in the largest gaps generated by GRAS-Di genotyping, we observed a slight increase of these largest gaps in some chromosomes (Figure 3, Table 3 and Table 4). Since the corresponding markers were in the same position as in linkage maps of GoldenGate assay, this slight increase was considered as a potential error of calculation. In some other chromosomal gap regions, despite the presence of additional markers incorporated after GRAS-Di genotyping, the largest gap did not much change, this is because the new markers are closely located to markers obtained by the GoldenGate assay.

Yamamoto and collaborators [11] sequenced the leading Japanese variety Koshihikari and found fewer SNP regions (compared with Nipponbare reference sequence) in chromosomes 5, 6, and 9, resulting in SNP gaps [36]. This was explained by the absence of DNA polymorphism between Koshihikari and Nipponbare because of the share of common chromosome segments on both genomes. This might also explain the absence of DNA polymorphism between Koshihikari and Yamadanishiki, Koshihikari and Taichung 65, Koshihikari and Fujisaka 5, and Koshihikari and Futaba [36]. However, in the present study, DNA polymorphisms were detected by NGS, which revealed a high possibility of DNA polymorphisms in regions where no DNA markers were obtained before.

Climate change and the unpredictable performance (for example, heading date and grain yield) of cereal crops are of increasing concern. Japanese rice population, which originated from closely related cultivars, has a narrow genetic diversity, which hampered previous temptations to perform genetic dissection of phenotypic variations observed among Japanese rice cultivars. To identify candidate genes or QTLs responsible for the phenotypic and performances differences between Japanese rice cultivars, we constructed RILs among four Japanese rice and Koshihikari cultivars, which are genetically close.

The present study used a recently developed method for genotyping by sequencing “GRAS-Di”, which was highly effective and could identify a large number of genetic markers despite the lack of DNA polymorphism between the used parent lines. The integration of markers generated by GRAS-Di and a previously generated GoldenGate SNP markers produced a higher density linkage map with significantly reduced average distance between markers. In addition to these genotyping data, the same RILs were used to assess several phenotypical traits, such as those related to the heading date. The combination of these phenotyping with the genotyping data generated by GRAS-Di allowed for the efficient mapping of QTLs controlling heading date in Japanese rice. Interestingly, the position of *Hd6* and *Hd16*, corresponding to two previously reported QTLs in chromosome 3 [24,39], was consistently confirmed in this study using all linkage maps generated by GRAS-Di. Likewise, the positions of three other QTLs reported in this study were also previously reported as QTLs located at the top and the middle of chromosome 6 (*Hd17* and *Hd1*, respectively) [40,41] and the end of chromosome 7 (*Hd2*, [42]). This result demonstrates that major QTLs for heading date, previously reported, could be successfully confirmed using linkage maps generated by GRAS-Di genotyping platform.

## 4. Materials and Methods

### 4.1. Plant Material and DNA Isolation

Four recombinant inbred lines (RIL71, RIL98, RIL16, and RIL91) of *O. sativa* subsp. *japonica*, developed from independent crosses of cultivars Yamadanishiki, Taichung 65, Fujisaka 5, and Futaba, respectively, with the common cultivar Koshihikari, were used in this study. The obtained F_1_ progeny was self-fertilized to produce F_2_ progeny. Each of the F_2_ was brought to F_7_ generation by a single seed descent (SSD) method to generate a final progeny number of 190, 96, 95, and 94 for RIL 71, RIL98, RIL16, and RIL91, respectively (Figure 1). These populations were developed in the experimental field at the Food Resources Education and Research Center, Graduate School of Agricultural Science, Kobe University, Kasai City, Hyogo Prefecture, Japan. Bulked leaf samples from each of the four RILs populations (F_7_ generation) and their respective parents were collected from one-month-old seedlings, dried overnight at 50 °C, and used for total genomic DNA extraction using CTAB [43] protocol with slight modifications.

### 4.2. DNA Sequencing and Genotyping

We used genotyping by random amplicon sequencing-direct (GRAD-Di) technology for sequencing and genotyping the four RIL populations and their respective parents. GRAS-Di has been recently developed by Toyota Motor Corporation [12] and licensed to Eurofins Genomics. This technology allows for the amplification of multiple parts of the genome by performing two rounds of PCR with high concentration of random primers and adapter sequences to generate a sequence library of tens of thousands of amplicons (Supplementary Figure S1A). GRAS-Di PCR first used a high concentration of random primers (Supplementary Table S1), with Nextera adaptor sequences and 3-base random oligomers that randomly bind to genomic DNA to amplify nonspecific regions of the entire genome. The second PCR is used for indexing and includes Illumina multiplexing 8-base dual index and P7/P5 adapter sequence (Supplementary Table S2). The final genome-wide amplified amplicons were pooled and purified for sequencing using either HiSeq2500 NGS-platforms (Illumina, Inc., San Diego, CA, USA) to generate paired-end reads ranging from 100 to 150 bp depending on the sequence platform (main steps for genotyping and construction of linkage maps are summarized in Supplementary Figure S1B). Construction of library and sequencing of the amplicons were carried out by Eurofins Genomics (Supplementary Figure S1A). To determine the optimal coverage to be used for efficient sequencing and to obtain as many SNP markers as possible, we performed a first sequencing trial of RIL71 using two independent replications of the same library (95 lines). When the sequence depth was 1× the genome, the number of obtained markers was 1089. However, when the depth increased to 2×the genome, the number of markers did not improve much (only 1792 markers were generated), which suggested that 1× sequencing depth should be adequate to detect SNP markers distributed throughout the genome.

For SNP and amplicons marker detection, adapter sequences were removed using cutadapt (ver.1.16) company software. Trimmed reads were mapped to Nipponbare (IRGSP-1.0) reference sequence using Bowtie2 (ver.2.3.3.1) software. For the detection of SNP markers, sequence bases that were different from the Nipponbare reference were extracted using samtools (ver.1.6)/bcftools (ver.1.6) and SNP mutations filtered using vcftools. Further filtering using beagle (ver.4.0) software was performed to ultimately keep only reliable mutations that enable detection of polymorphism between RILs and their corresponding parents. For convenience, SNPs were named on the basis of their physical locations (SNP/chromosome number/physical position). For amplicon markers detection, the polymorphism was judged on the basis of the presence or absence of a particular sequence read in the genome of both parents (i.e., the presence of a sequence in one parent genome and absence in the second parent genome). Once defined as a reliable amplicon marker to be used for genotyping, each RILs population was genotyped on the basis of the presence or absence of the read, and the genomic position of the amplicon marker was determined using the “Nipponbare” (IRGSP-1.0) reference sequence. The analysis of the obtained results was characterized by high reproducibility of amplicon amplification and a minimal loss of genotype data. Amplicon markers were named as follows: AMP/chromosome number/physical position.

SNP marker identification and genotyping using GoldenGate assay were performed using procedures described by Yamamoto and collaborators [11]. Candidate SNPs between Nipponbare and Koshihikari genome sequences were selected at a spacing of 100 to 200 kb and used for genotyping. SNP adaptability to the Illumina (San Diego, CA, USA) GoldenGate detection system was scored using the Illumina online scoring system (http://icom.illumina.com). SNPs with a score higher than 0.4 were selected to design 768-plex SNPs for Illumina GoldenGate BeadArray technology platform [11]. A total of 151 representative Japanese rice cultivars subsp *japonica*, including Yamadanishiki, Taichung 65, Fujisaka 5, and Futaba, which have been grown during the past 150 years, were used for SNP array analysis. Total DNA from parents of four RIL populations was extracted, and 5µL of 50 ng/µL DNA was used for SNP analysis. Data processing and bioinformatics analysis were conducted by Eurofin Genomics. To check the coherence between markers obtained by GoldenGate and GRAS-Di genotyping, the same genomic regions of all markers obtained by GoldenGate and GRAS-Di were compared on the basis of a threshold of 50 kb distance between GoldenGate-SNP and GRAS-Di-amplicon, or GoldenGate-SNP and GRAS-Di-SNP. Red cell color for each DNA marker corresponds to the genomic region of Koshihikari (A) and green cell color corresponds to the founder genotype (B). As shown in the tables of Supplementary Figure S3, almost all markers detected by GoldenGate and having A or B genotypes have been confirmed with the same genotype using GRAS-Di technology. The corresponding ratio is the quotient of the sum of markers having the same genotype regardless of the genotyping method to the total number of markers generated by GRAS-DI technology.

### 4.3. Linkage Map Construction

Following the alignment of sequence reads to the Nipponbare (IRGSP-1.0 [44]) reference sequence and after filtering SNPs and sorting them according to their genomic position, a quality control of the genotyping data was performed to exclude any possible alignment errors. Markers showing only Koshihikari or second parent founder homozygous or heterozygous genotypes were discarded. Too many heterozygous markers were also excluded from the analysis. Physically closely located markers that still show recombination are unlikely to be true markers and, thus, were excluded from the analysis. Ambiguous genotypes, for instance, ambiguous amplicon markers mapped to Nipponbare reference sequence with low read count, were converted to missing genotypes codes NA (not applicable) or “-“. Within a set of co-localized markers, only one reliable marker was kept, and other co-localized markers were discarded. SNP markers confirmed with a ratio of 1:1 (Koshihikari:Founder) were used to construct the genetic linkage map. Kosambi’s mapping function was used to convert the recombination frequency to a genetic map distance [45]. The R package R/qtl was used to display the linkage map [46].

### 4.4. Evaluation of Phenotypic Data and QTL Analysis

In 2021, the four RIL populations and their parents were sown from April 26th to 28th. Six plants per line were transplanted to the experimental field at Kobe University, Food Resources Education and Research Center (Kasai City, Hyogo Prefecture, Japan; 34.88 N, 134.86 E) between June 1st and June 2nd of the same year. The evaluation of days to heading was conducted on six plants per line. QTL analysis was performed using linkage maps derived from the four RIL populations using the R package R/qtl [46]. Detection of QTLs was carried out using the composite interval mapping method [47] with a setting of window size and walk speed of 3 and 1 cM, respectively. The empirical threshold logarithm of odds (LOD) values were determined by computing 1000 permutations [48]. Confidence intervals were calculated from the 1-LOD support interval.

## Figures and Tables

**Table 1:** Summary of whole-genome sequencing data obtained for GRAS-Di genotyping of the four RIL populations.

	Called Bases (Mbp)	Q30(%)	Average Quality	Mapping Ratio to IRGSP-1.0 (%)
RIL71-2nd (95 lines)	438.7	92.9	35.3	97.5
Koshihikari	413.5	92.8	35.3	98.0
Yamadanishiki	457.0	92.8	35.3	98.3
RIL16 (95 lines)	440.0	92.3	35.1	97.3
Koshihikari	413.5	92.8	35.3	98.0
Fujisaka 5	487.0	92.3	35.1	98.1
RIL91 (94 lines)	403.1	92.7	35.2	98.3
Koshihikari	413.5	92.8	35.3	98.0
Futaba	439.0	92.8	35.2	98.3
RIL98 (96 lines)	392.0	93.1	35.3	96.1
Koshihikari	413.5	92.8	35.3	98.0
Taichung 65	373.0	93.0	35.3	98.4

**Table 2:** Details on linkage maps of four RIL populations RIL71 (A), RIL98 (B), RIL16 (C) and RIL91 (D), integrating both markers generated by GoldenGate system and GRAS-Di technology, excluding the co-localized markers.

**(A) RIL71**							
**Chr.**	**No of Markers**	**Total Length (cM)**	**Marker Interval (cM)**		**Marker Name**	**Physical Position (Mb)**	**physical Distance (Mb)**
			**Average Distance**	**Largest Gap**			
1	69	202	3	29.9	SNP1-6	4.93	3.11
					ac01000670	8.04	
2	59	158.3	2.7	16.9	SNP2-29	22.56	3.03
					aa02002928	25.59	
3	40	157.3	4	28.8	aa03000857	12.88	3.85
					SNP3-28	16.72	
4	47	131.2	2.9	28.9	ac04000676	16.74	3.32
					SNP4-46	20.06	
5	29	132.5	4.7	36.6	ac05000011	0.46	3.43
					aa05000263	3.89	
6	51	121.4	2.4	14	ac06000665	18.89	2.44
					AMP0074317	21.32	
7	51	116.2	2.3	10	aa07001816	5.21	1.98
					aa07001842	7.18	
8	37	116.3	3.2	20	SNP8-28	10.55	8.9
					aa08005473	19.45	
9	23	85.8	3.9	18	AMP0066980	13.03	3.09
					ac09000278	16.12	
10	34	81.7	2.5	18	ac10000399	15.13	3.42
					ac10000429	18.55	
11	47	113	2.5	11.1	SNP11-34	18.74	1.61
					aa11004155	20.35	
12	40	99.3	2.5	8.5	aa12005168	24.56	1.38
					SNP12-32	25.93	
Total	527	1515	2.9	-			
**(B) RIL98**							
**Chr.**	**No of Markers**	**Total Length (cM)**	**Marker Interval (cM)**		**Marker Name**	**Physical Position (Mb)**	**Physical Distance (Mb)**
			**Average Distance**	**Largest Gap**			
1	48	200.4	4.3	36	AMP0078803	4.99	5.74
					SNP01-16	10.73	
2	48	141.3	3	16.9	ab02000190	6.52	3.08
					aa02000772	9.6	
3	41	156.5	3.9	27.8	SNP03-23	12.5	4.22
					SNP03-24	16.72	
4	32	117.8	3.8	27.5	SNP04-40	13.99	4.63
					AMP0036911	18.61	
5	29	114.2	4.1	17.8	ab05000280	22.81	4.03
					aa05000868	26.84	
6	38	128	3.5	22.4	ac06000665	18.89	3.9
					AMP0001588	22.79	
7	52	109.4	2.1	9	aa07007512	28.29	0.77
					aa07007522	29.06	
8	40	106.1	2.7	17.2	aa08006250	21.73	3.11
					ab08000934	24.84	
9	19	90.4	5	16.5	SNP09-4	14.82	1.29
					ac09000278	16.12	
10	25	106.9	4.5	23	AMP0074848	3.56	2.44
					AMP0028902	6	
11	39	113.6	3	15.1	aa11004053	18.05	2.3
					aa11004155	20.35	
12	44	104.5	2.4	12.5	AMP0021554	0	2.09
					aa12000015	2.09	
Total	455	1489	3.4	-			
**(C) RIL16**							
**Chr.**	**No of Markers**	**Total Length (cM)**	**Marker Interval (cM)**		**Marker Name**	**Physical Position (Mb)**	**physical Distance (Mb)**
			**Average Distance**	**Largest Gap**			
1	63	195.7	3.2	23	SNP1-18	6.77	2.65
					ab01000593	9.42	
2	50	160.1	3.3	25.7	aa02000707	5.6	4.05
					SNP2-6	9.65	
3	46	158	3.5	37.8	AMP0032478	8.53	8.2
					AMP0019926	16.72	
4	41	108.4	2.7	29.4	AMP0016137	23.02	8.38
					aa04008763	31.41	
5	27	143.3	5.5	36.9	ab05000017	1.78	3.91
					AMP0033587	5.69	
6	48	100.3	2.1	11.1	AMP0031636	23.56	1.77
					SNP6-64	25.33	
7	45	114.3	2.6	10.3	AMP0016664	24.67	1.97
					aa07007162	26.64	
8	35	111.5	3.3	25.7	aa08006250	21.73	5.74
					SNP8-47	27.47	
9	35	98.3	2.9	11.9	SNP9-26	9.53	1.62
					SNP9-30	11.15	
10	32	88.8	2.9	9.2	aa10000871	2.81	1.18
					aa10000954	3.99	
11	47	120.2	2.6	9.5	SNP11-25	18.74	1.61
					aa11004155	20.35	
12	32	86.3	2.8	15.4	aa12004743	21.48	3.41
					AMP0028108	24.9	
Total	501	1485.2	3	-			
**(D) RIL91**							
**Chr.**	**No of Markers**	**Total Length (cM)**	**Marker Interval (cM)**		**Marker Name**	**Physical Position (Mb)**	**physical Distance (Mb)**
			**Average Distance**	**Largest Gap**			
1	57	183.6	3.3	30.9	AMP0022274	4.97	3.06
					ac01000670	8.04	
2	51	138.8	2.8	16.4	aa02000715	6.1	3.49
					aa02000772	9.6	
3	41	150.5	3.8	35.8	SNP3-5	7.39	8.56
					ab03000375	15.96	
4	32	145.5	4.7	51.2	SNP4-39	23.27	8.25
					ab04001335	31.52	
5	25	107.2	4.5	33.7	SNP5-1	0.02	5.68
					AMP0033375	5.69	
6	39	117.6	3.1	14.5	SNP6-36	23.66	1.57
					SNP6-39	25.23	
7	32	113.7	3.7	17.9	aa07003357	22.25	4.62
					SNP7-22	26.87	
8	38	98.8	2.7	19.3	aa08006250	21.73	4.36
					ab08000952	26.09	
9	23	79.1	3.6	11.7	ab09001035	16.58	3.03
					SNP9-13	19.61	
10	28	76.8	2.8	9.7	aa10003142	16.8	1.68
					SNP10-23	18.48	
11	41	104.6	2.6	13.9	aa11004053	18.05	2.3
					aa11004155	20.35	
12	29	73.7	2.6	8.7	SNP12-42	25.86	1.3
					SNP12-43	27.16	
Total	436	1389.9	3.35	-			

**Table 3:** Details on integration linkage maps of four RIL populations RIL71 (A), RIL98 (B), RIL16 (C) and RIL91 (D) including both markers generated by GoldenGate system and GRAS-Di technology (including the co-localized markers).

**(A) RIL71**							
**Chr.**	**No of Markers**	**Total Length (cM)**	**Marker Interval (cM)**		**Marker Name**	**Physical Position (Mb)**	**Physical Distance (Mb)**
			**Average Distance**	**Largest Gap**			
1	150	202.1	1.4	29.9	SNP1-13	4.95	3.09
					ac01000670	8.04	
2	111	158.5	1.4	16.9	SNP2-29	22.56	3.03
					aa02002928	25.59	
3	87	157.3	1.8	28.8	aa03000857	12.88	3.85
					SNP3-28	16.72	
4	152	131.2	0.9	28.9	ac04000676	16.74	3.32
					SNP4-46	20.06	
5	84	132.6	1.6	36.6	ac05000011	0.46	3.43
					aa05000263	3.89	
6	144	122.2	0.9	13.9	ac06000665	18.89	2.44
					AMP0074317	21.32	
7	175	116.8	0.7	10	aa07001816	5.21	1.98
					aa07001842	7.18	
8	91	116.4	1.3	20	SNP8-28	10.55	8.9
					aa08005473	19.45	
9	31	85.8	2.9	18	AMP0066980	13.03	3.09
					ac09000278	16.12	
10	100	82.8	0.8	18	ac10000399	15.13	3.42
					ac10000429	18.55	
11	119	113.7	1	11.1	SNP11-34	18.74	1.61
					aa11004155	20.35	
12	116	99.4	0.9	8.5	SNP12-31	24.56	1.37
					AMP0016171	25.93	
Total	1360	1518.8	1.1	-			
**(B) RIL98**							
**Chr.**	**No of Markers**	**Total Length (cM)**	**Marker Interval (cM)**		**Marker Name**	**Physical Position (Mb)**	**physical Distance (Mb)**
			**Average Distance**	**Largest Gap**			
1	142	201.5	1.4	36	AMP0078803	4.99	5.74
					AMP0091552	10.73	
2	137	141.4	1	16.9	ac02000121	6.59	3.01
					aa02000772	9.6	
3	100	156.6	1.6	27.8	SNP03-23	12.5	4.22
					SNP03-24	16.72	
4	174	118.4	0.7	27.5	SNP04-40	13.99	4.63
					AMP0036911	18.61	
5	68	114.2	1.7	17.8	ac05000298	23.22	3.62
					aa05000868	26.84	
6	122	128.2	1.1	22.6	ac06000665	18.89	3.9
					AMP0001588	22.79	
7	281	110.5	0.4	9	aa07007512	28.29	0.77
					aa07007522	29.06	
8	155	106.1	0.7	17.2	aa08006250	21.73	3.11
					ab08000934	24.84	
9	30	90.4	3.1	16.5	SNP09-4	14.82	1.29
					ac09000278	16.12	
10	90	108	1.2	23	AMP0074848	3.56	2.42
					AMP0027374	5.98	
11	153	113.7	0.7	15.1	aa11004053	18.05	2.3
					aa11004155	20.35	
12	153	104.8	0.7	12.5	AMP0021554	0	2.09
					aa12000015	2.09	
Total	1605	1493.9	0.9	-			
**(C) RIL16**							
**Chr.**	**No of Markers**	**Total Length (cM)**	**Marker Interval (cM)**		**Marker Name**	**Physical Position (Mb)**	**physical Distance (Mb)**
			**Average Distance**	**Largest Gap**			
1	188	196.6	1.1	23	SNP1-18	6.77	2.54
					AMP0005441	9.32	
2	120	161	1.4	25.7	aa02000707	5.6	4
					aa02000772	9.6	
3	163	159.6	1	37.8	AMP0032478	8.53	8.2
					AMP0019926	16.72	
4	208	109.2	0.5	29.5	SNP4-56	23.27	8.14
					aa04008763	31.41	
5	65	143.4	2.2	36.9	SNP5-2	1.78	3.91
					AMP0033587	5.69	
6	220	102.6	0.5	11.1	SNP6-62	23.66	1.57
					AMP0025701	25.23	
7	352	114.4	0.3	10.3	AMP0016664	24.67	1.97
					aa07007162	26.64	
8	148	111.6	0.8	25.7	aa08006250	21.73	5.74
					AMP0028208	27.47	
9	159	99.1	0.6	11.9	AMP0005465	9.94	1.21
					SNP9-30	11.15	
10	79	88.8	1.1	9.2	aa10000871	2.81	1.18
					aa10000954	3.99	
11	218	120.2	0.6	8.9	SNP11-25	18.74	1.43
					SNP11-26	20.16	
12	98	87.5	0.9	15.4	aa12004743	21.48	3.41
					AMP0028107	24.9	
Total	2018	1493.9	0.7	-			
**(D) RIL91**							
**Chr.**	**No of Markers**	**Total Length (cM)**	**Marker Interval (cM)**		**Marker Name**	**Physical Position (Mb)**	**physical Distance (Mb)**
			**Average Distance**	**Largest Gap**			
1	172	183.8	1.1	30.9	AMP0022274	4.972	3.06
					ac01000670	8.036	
2	156	141.5	0.9	16.4	aa02000715	6.104	3.49
					aa02000772	9.597	
3	100	150.7	1.5	35.8	SNP3-5	7.393	8.56
					ab03000375	15.958	
4	169	145.5	0.9	51	SNP4-39	23.269	8.14
					aa04008763	31.407	
5	82	107.6	1.3	33.7	aa05000026	0.164	5.53
					AMP0033375	5.694	
6	159	117.7	0.7	14.5	SNP6-38	23.663	1.57
					AMP0025619	25.229	
7	90	114.2	1.3	17.9	aa07003357	22.25	4.39
					aa07007162	26.641	
8	515	98.8	0.2	19.3	aa08006250	21.733	4.36
					ab08000952	26.093	
9	70	79.2	1.1	11.8	ab09001035	16.582	2.72
					AMP0005719	19.305	
10	101	77.5	0.8	9.7	aa10003172	17.125	1.36
					AMP0015172	18.481	
11	280	104.6	0.4	13.9	aa11004053	18.048	2.3
					aa11004155	20.347	
12	162	74	0.5	8.8	AMP0017302	25.986	1.17
					AMP0015946	27.159	
Total	2,056	1395.2	0.7	-			1

**Table 4:** A linkage map created using markers obtained by the GoldenGate method, excluding the co-localized markers.

**(A) RIL71**							
**Chr.**	**No of Markers**	**Total Length (cM)**	**Marker Interval (cM)**		**Marker Name**	**Physical Position (Mb)**	**physical Distance (Mb)**
			**Average Distance**	**Largest Gap**			
1	45	195.6	4.4	30.2	aa01005142	4.84	3.2
					ac01000670	8.04	
2	29	163.2	5.8	18	aa02001544	22.53	3.06
					aa02002928	25.59	
3	26	159.8	6.4	30.7	aa03000857	12.88	4.48
					ac03000493	17.36	
4	23	132	6	31.1	ac04000676	16.74	3.79
					aa04007763	20.53	
5	19	130.2	7.2	34.9	ac05000011	0.46	3.43
					aa05000263	3.89	
6	23	116.7	5.3	16.8	ac06000103	6.09	2.57
					ac06000385	8.66	
7	24	115.5	5	23.7	aa07001934	19.24	4.47
					aa07005205	23.71	
8	16	112.8	7.5	23.7	aa08001560	8.84	10.62
					aa08005473	19.45	
9	15	83.6	6	24.7	ac09000231	11.75	4.36
					ac09000278	16.12	
10	14	80.5	6.2	17.9	ac10000399	15.13	3.42
					ac10000429	18.55	
11	29	112.7	4	13.7	aa11004053	18.05	2.3
					aa11004155	20.35	
12	29	94.8	3.4	11.2	aa12004649	17.48	2.38
					aa12004709	19.86	
Total	292	1497.3	5.3	-			
**(B) RIL98**							
**Chr.**	**No of Markers**	**Total Length (cM)**	**Marker Interval (cM)**		**Marker Name**	**Physical Position (Mb)**	**Physical Distance (Mb)**
			**Average Distance**	**Largest Gap**			
1	32	204.1	6.6	41	aa01005142	4.84	6.13
					aa01005640	10.97	
2	24	135.9	5.9	16.8	ab02000190	6.52	3.08
					aa02000772	9.6	
3	31	160.4	5.3	43.4	ac03000229	9.29	8.07
					ac03000493	17.36	
4	17	99.4	6.2	35.3	aa04003679	7.79	13.73
					ac04001045	21.53	
5	18	122.3	7.2	20.1	ab05000280	22.81	4.03
					aa05000868	26.84	
6	21	128.8	6.4	38.5	ac06000665	18.89	6.63
					aa06000938	25.52	
7	28	106.9	4	19.4	aa07002141	20.25	2.99
					aa07005154	23.24	
8	24	106.9	4.6	17.3	aa08006250	21.73	3.11
					ab08000934	24.84	
9	13	80.2	6.7	33.5	ac09000238	12.73	3.39
					ac09000278	16.12	
10	15	115.9	8.3	47.2	aa10000749	2.15	9.82
					aa10002652	11.97	
11	27	109.9	4.2	16.4	aa11004053	18.05	2.3
					aa11004155	20.35	
12	27	94.6	3.6	10.6	aa12004649	17.48	1.65
					aa12004670	19.14	
Total	277	1465.2	5.5	-			
**(C) RIL16**							
**Chr.**	**No of Markers**	**Total Length (cM)**	**Marker Interval (cM)**		**Marker Name**	**Physical Position (Mb)**	**physical Distance (Mb)**
			**Average Distance**	**Largest Gap**			
1	35	193.2	5.7	30.9	ac01000635	6.34	3.08
					ab01000593	9.42	
2	30	165.9	5.7	25.5	aa02000707	5.6	4
					aa02000772	9.6	
3	27	184.4	7.1	63.7	ab03000111	8.2	10.26
					aa03002110	18.46	
4	23	99.8	4.5	25.8	ab04001157	23.13	8.28
					aa04008763	31.41	
5	16	135.4	9	54.1	ab05000017	1.78	10.62
					ab05000128	12.4	
6	21	92.4	4.6	26.7	ac06000669	19.78	6.57
					aa06001093	26.35	
7	22	117.1	5.6	22.7	aa07005205	23.71	2.93
					aa07007162	26.64	
8	21	92.3	4.6	31.7	aa08000774	2.23	2.85
					aa08000792	5.08	
9	14	96.2	7.4	46.5	aa09000038	9.07	7.04
					ac09000278	16.12	
10	18	89	5.2	12.2	ac10000003	0.06	2.75
					aa10000871	2.81	
11	21	122.3	6.1	20.3	aa11004155	20.35	3.27
					aa11005083	23.61	
12	14	74.4	5.7	21.6	aa12000100	2.84	8.93
					aa12004439	11.77	
Total	262	1462.4	5.8	-			
**(D) RIL91**							
**Chr.**	**No of Markers**	**Total Length (cM)**	**Marker Interval (cM)**		**Marker Name**	**Physical Position (Mb)**	**physical Distance (Mb)**
			**Average Distance**	**Largest Gap**			
1	39	168.4	4.4	25.4	aa01005142	4.84	3.2
					ac01000670	8.04	
2	36	132.7	3.8	16.8	aa02000715	6.1	3.49
					aa02000772	9.6	
3	27	137.2	5.3	30.7	aa03002463	29.09	3.89
					ab03000579	32.98	
4	24	143	6.2	49.1	ab04001157	23.13	8.28
					aa04008763	31.41	
5	15	88.6	6.3	38.8	aa05000007	0.03	12.37
					ab05000128	12.4	
6	22	116.8	5.6	19.3	ac06000764	21.7	3.82
					aa06000938	25.52	
7	25	105.4	4.4	17.9	aa07003357	22.25	4.39
					aa07007162	26.64	
8	24	95.7	4.2	18.2	aa08000774	2.23	2.85
					aa08000792	5.08	
9	10	72.3	8	23.3	ab09001035	16.58	5.05
					aa09000103	21.63	
10	18	75.9	4.5	11.2	aa10003172	17.12	1.43
					ac10000429	18.55	
11	30	103.7	3.6	14	aa11004053	18.05	2.3
					aa11004155	20.35	
12	16	65.5	4.4	11.9	aa12000100	2.84	2.69
					aa12001794	5.53	
Total	286	1305.1	4.8	-			

## Data Availability

Not applicable.

## Supplementary Materials

The following supporting information can be downloaded at: https://www.mdpi.com/article/10.3390/plants12040929/s1, Figure S1. (A) Scheme summarizing the main steps for GRAS-Di library construction in this work. (B) Flow chart of main steps adopted for genotyping analysis and linkage mapping; Table S1. Primer sequences for the first PCR; Table S2. Primer sequences for the second PCR; Figure S2: Total number of markers generated by GRAS-Di; Figure S3: Example of calculation of correspondence ratio between makers generated by GoldenGate method and markers generated by GRAS-Di technology.
