# Supplementary material for: High-Density Linkage Maps from Japanese Rice japonica Recombinant Inbred Lines Using Genotyping by Random Amplicon Sequencing-Direct (GRAS-Di)
Source: Plants (Basel). 2023 Feb 17;12(4):929. doi: 10.3390/plants12040929 (PMC9959243; doi:10.3390/plants12040929)
Supplement: Supplementary file 1 [file plants-12-00929-s001.zip › plants-2185407-supplementary.pdf]

(A)

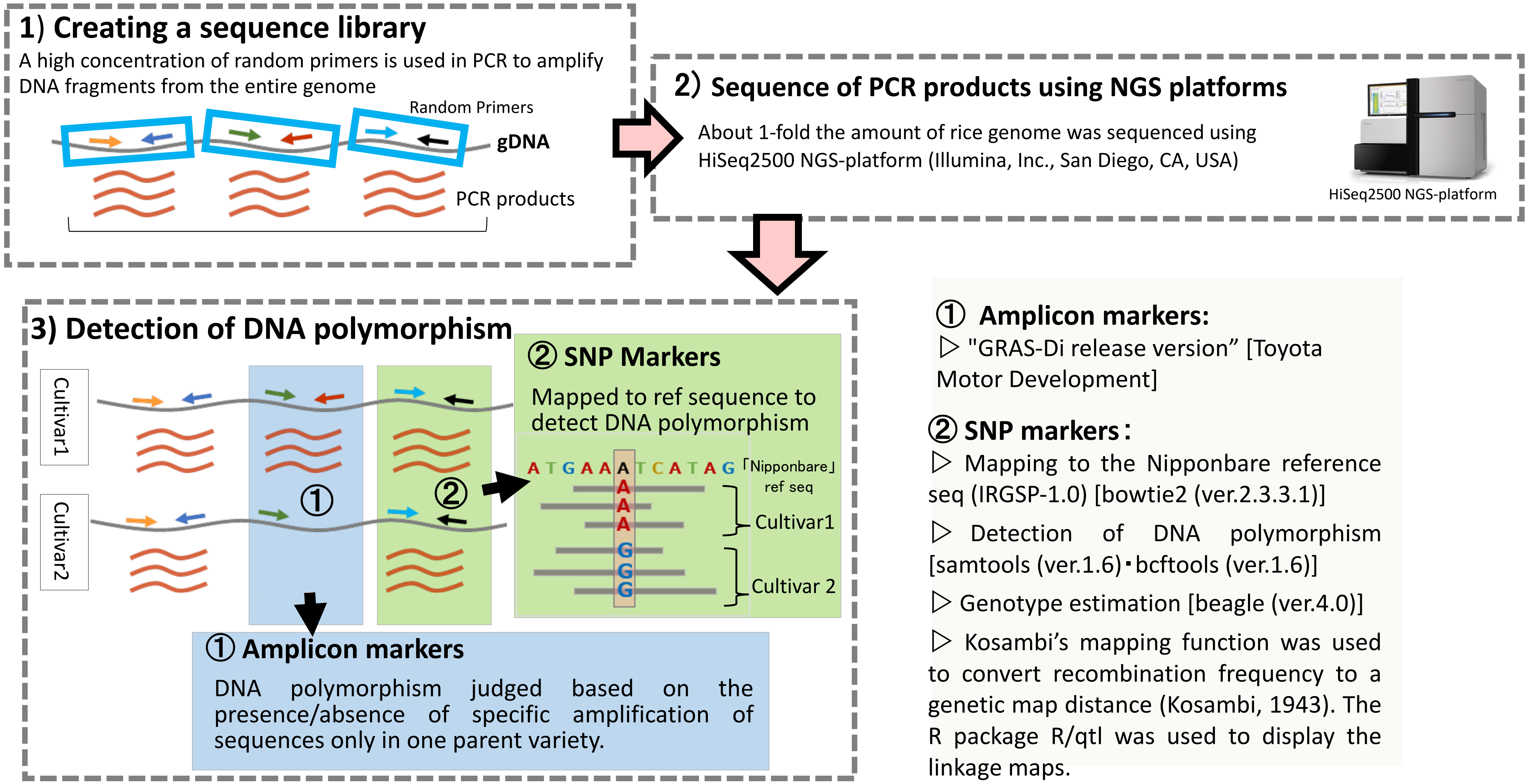

1) Creating a sequence library
A high concentration of random primers is used in PCR to amplify DNA fragments from the entire genome
Random Primers
gDNA
PCR products
2) Sequence of PCR products using NGS platforms
About 1-fold the amount of rice genome was sequenced using HiSeq2500 NGS-platform (Illumina, Inc., San Diego, CA, USA)
HiSeq2500 NGS-platform
3) Detection of DNA polymorphism
Cultivar1
Cultivar2
①
②
② SNP Markers
Mapped to ref sequence to detect DNA polymorphism
A T G A A A T C A T A G
「Nipponbare」 ref seq
A
A
A
Cultivar1
G
G
G
Cultivar 2
① Amplicon markers
DNA polymorphism judged based on the presence/absence of specific amplification of sequences only in one parent variety.
① Amplicon markers:
▷ "GRAS-Di release version" [Toyota Motor Development]
② SNP markers:
▷ Mapping to the Nipponbare reference seq (IRGSP-1.0) [bowtie2 (ver.2.3.3.1)]
▷ Detection of DNA polymorphism [samtools (ver.1.6)・bcftools (ver.1.6)]
▷ Genotype estimation [beagle (ver.4.0)]
▷ Kosambi's mapping function was used to convert recombination frequency to a genetic map distance (Kosambi, 1943). The R package R/qtl was used to display the linkage maps.

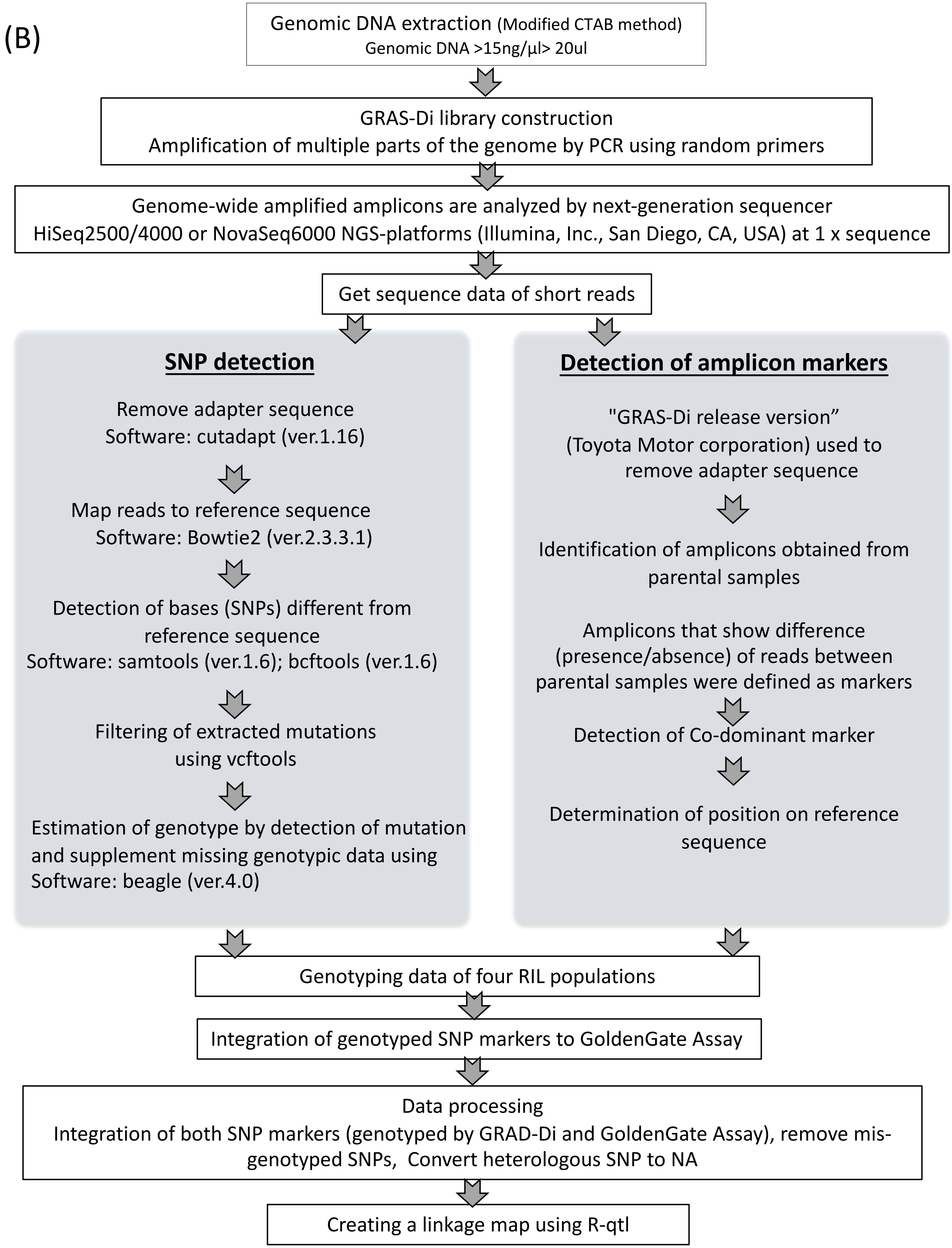

Figure S1. (A) Scheme summarizing the main steps for GRAS-Di library construction in this work . (B) Flow chart of main steps adopted for genotyping analysis and linkage mapping

Table S1. Primer sequences for the first PCR

Primer Name	Primer Sequence (5'-3')
NE10_301	TAAGAGACAGAAA
NE10_302	TAAGAGACAGAAC
NE10_303	TAAGAGACAGAAG
NE10_304	TAAGAGACAGAAT
NE10_305	TAAGAGACAGACA
NE10_306	TAAGAGACAGACC
NE10_307	TAAGAGACAGACG
NE10_308	TAAGAGACAGACT
NE10_309	TAAGAGACAGAGA
NE10_310	TAAGAGACAGAGC
NE10_311	TAAGAGACAGAGG
NE10_312	TAAGAGACAGAGT
NE10_313	TAAGAGACAGATA
NE10_314	TAAGAGACAGATC
NE10_315	TAAGAGACAGATG
NE10_316	TAAGAGACAGATT
NE10_317	TAAGAGACAGCAA
NE10_318	TAAGAGACAGCAC
NE10_319	TAAGAGACAGCAG
NE10_320	TAAGAGACAGCAT
NE10_321	TAAGAGACAGCCA
NE10_322	TAAGAGACAGCCC
NE10_323	TAAGAGACAGCCG
NE10_324	TAAGAGACAGCCT
NE10_325	TAAGAGACAGCGA
NE10_326	TAAGAGACAGCGC
NE10_327	TAAGAGACAGCGG
NE10_328	TAAGAGACAGCGT
NE10_329	TAAGAGACAGCTA
NE10_330	TAAGAGACAGCTC
NE10_331	TAAGAGACAGCTG
NE10_332	TAAGAGACAGCTT
NE10_333	TAAGAGACAGGAA
NE10_334	TAAGAGACAGGAC
NE10_335	TAAGAGACAGGAG
NE10_336	TAAGAGACAGGAT
NE10_337	TAAGAGACAGGCA
NE10_338	TAAGAGACAGGCC
NE10_339	TAAGAGACAGGCG
NE10_340	TAAGAGACAGGCT
NE10_341	TAAGAGACAGGGA
NE10_342	TAAGAGACAGGGC
NE10_343	TAAGAGACAGGGG
NE10_344	TAAGAGACAGGGT
NE10_345	TAAGAGACAGGTA
NE10_346	TAAGAGACAGGTC
NE10_347	TAAGAGACAGGTG
NE10_348	TAAGAGACAGGTT
NE10_349	TAAGAGACAGTAA
NE10_350	TAAGAGACAGTAC
NE10_351	TAAGAGACAGTAG
NE10_352	TAAGAGACAGTAT
NE10_353	TAAGAGACAGTCA
NE10_354	TAAGAGACAGTCC
NE10_355	TAAGAGACAGTCG
NE10_356	TAAGAGACAGTCT
NE10_357	TAAGAGACAGTGA
NE10_359	TAAGAGACAGTGG
NE10_360	TAAGAGACAGTGT
NE10_361	TAAGAGACAGTTA
NE10_362	TAAGAGACAGTTC
NE10_363	TAAGAGACAGTTG
NE10_364	TAAGAGACAGTTT

Table S2. Primer sequences for the second PCR

**GRAS-Di library construct（Illumina Nextera adapter sequnece used）**
(P5 side) 5'-AATGATACGGCGACCACCGAGATCTACAC[P5 index sequence(below)]TCGTCGGCAGCGTCAGATGTGTATAAGAGACAG[Insert]CTGTCTCTTATACACATCTCCGAGCCCACGAGAC[P7 index sequence(below)]ATCTCGTATGCCGTCTTCTGCTTG-3' (P7 side)

P5 Index Name	Index Primer Sequence (5'-3')	Index Sequence
P5_0001	AATGATACGGCGACCACCGAGATCTACACCGCGCAGATCGTCGGCAGCGTCAGATGTGTATAAGAGACAG	CGCGCAGA
P5_0002	AATGATACGGCGACCACCGAGATCTACACCGTCAGCATCGTCGGCAGCGTCAGATGTGTATAAGAGACAG	CGTCAGCA
P5_0003	AATGATACGGCGACCACCGAGATCTACACAGCGTCGATCGTCGGCAGCGTCAGATGTGTATAAGAGACAG	AGCGTCGA
P5_0004	AATGATACGGCGACCACCGAGATCTACACCATATGTATCGTCGGCAGCGTCAGATGTGTATAAGAGACAG	CATATGTA
P5_0005	AATGATACGGCGACCACCGAGATCTACACCAGACAGATCGTCGGCAGCGTCAGATGTGTATAAGAGACAG	CAGACAGA
P5_0007	AATGATACGGCGACCACCGAGATCTACACACTCACATTCGTCGGCAGCGTCAGATGTGTATAAGAGACAG	ACTCACAT
P5_0008	AATGATACGGCGACCACCGAGATCTACACGCTCTAGTTCGTCGGCAGCGTCAGATGTGTATAAGAGACAG	GCTCTAGT
P5_0009	AATGATACGGCGACCACCGAGATCTACACGCGCTCATTCGTCGGCAGCGTCAGATGTGTATAAGAGACAG	GCGCTCAT
P5_0010	AATGATACGGCGACCACCGAGATCTACACAGAGTAGATCGTCGGCAGCGTCAGATGTGTATAAGAGACAG	AGAGTAGA
P5_0011	AATGATACGGCGACCACCGAGATCTACACTATGAGCATCGTCGGCAGCGTCAGATGTGTATAAGAGACAG	TATGAGCA
P5_0012	AATGATACGGCGACCACCGAGATCTACACTCACTGATTCGTCGGCAGCGTCAGATGTGTATAAGAGACAG	TCACTGAT
P5_0013	AATGATACGGCGACCACCGAGATCTACACCTGACAGTTCGTCGGCAGCGTCAGATGTGTATAAGAGACAG	CTGACAGT
P5_0014	AATGATACGGCGACCACCGAGATCTACACGCGAGCTATCGTCGGCAGCGTCAGATGTGTATAAGAGACAG	GCGAGCTA
P5_0015	AATGATACGGCGACCACCGAGATCTACACCATATCGTTCGTCGGCAGCGTCAGATGTGTATAAGAGACAG	CATATCGT
P5_0016	AATGATACGGCGACCACCGAGATCTACACTCAGCAGTTCGTCGGCAGCGTCAGATGTGTATAAGAGACAG	TCAGCAGT
P5_0017	AATGATACGGCGACCACCGAGATCTACACGACATGTGTCGTCGGCAGCGTCAGATGTGTATAAGAGACAG	GACATGTG
P5_0018	AATGATACGGCGACCACCGAGATCTACACTGTATGAGTCGTCGGCAGCGTCAGATGTGTATAAGAGACAG	TGTATGAG
P5_0019	AATGATACGGCGACCACCGAGATCTACACCAGATCAGTCGTCGGCAGCGTCAGATGTGTATAAGAGACAG	CAGATCAG
P5_0003A	AATGATACGGCGACCACCGAGATCTACACTGCATAGATCGTCGGCAGCGTCAGATGTGTATAAGAGACAG	TGCATAGA
P5_0006A	AATGATACGGCGACCACCGAGATCTACACTGAGTCTATCGTCGGCAGCGTCAGATGTGTATAAGAGACAG	TGAGTCTA
P5_0007A	AATGATACGGCGACCACCGAGATCTACACGAGTACAGTCGTCGGCAGCGTCAGATGTGTATAAGAGACAG	GAGTACAG
P5_0008A	AATGATACGGCGACCACCGAGATCTACACTATACTGATCGTCGGCAGCGTCAGATGTGTATAAGAGACAG	TATACTGA
P5_0010A	AATGATACGGCGACCACCGAGATCTACACCATGTACATCGTCGGCAGCGTCAGATGTGTATAAGAGACAG	CATGTACA
P5_0011A	AATGATACGGCGACCACCGAGATCTACACTACTCGCTTCGTCGGCAGCGTCAGATGTGTATAAGAGACAG	TACTCGCT
P5_0012A	AATGATACGGCGACCACCGAGATCTACACCACTGTGTTCGTCGGCAGCGTCAGATGTGTATAAGAGACAG	CACTGTGT
P5_0013A	AATGATACGGCGACCACCGAGATCTACACTATGTAGTTCGTCGGCAGCGTCAGATGTGTATAAGAGACAG	TATGTAGT
P5_0014A	AATGATACGGCGACCACCGAGATCTACACGTGAGATGTCGTCGGCAGCGTCAGATGTGTATAAGAGACAG	GTGAGATG
P5_0015A	AATGATACGGCGACCACCGAGATCTACACTAGACTAGTCGTCGGCAGCGTCAGATGTGTATAAGAGACAG	TAGACTAG
P5_0016A	AATGATACGGCGACCACCGAGATCTACACGTGTCAGTTCGTCGGCAGCGTCAGATGTGTATAAGAGACAG	GTGTCAGT
P5_0018A	AATGATACGGCGACCACCGAGATCTACACTGAGCTATTCGTCGGCAGCGTCAGATGTGTATAAGAGACAG	TGAGCTAT
P5_0019A	AATGATACGGCGACCACCGAGATCTACACCAGCGTGATCGTCGGCAGCGTCAGATGTGTATAAGAGACAG	CAGCGTGA
P5_0020A	AATGATACGGCGACCACCGAGATCTACACCACGAGAGTCGTCGGCAGCGTCAGATGTGTATAAGAGACAG	CACGAGAG
P5_0021A	AATGATACGGCGACCACCGAGATCTACACCACTGATATCGTCGGCAGCGTCAGATGTGTATAAGAGACAG	CACTGATA
P5_0022A	AATGATACGGCGACCACCGAGATCTACACGCGTATATTCGTCGGCAGCGTCAGATGTGTATAAGAGACAG	GCGTATAT
P5_0023A	AATGATACGGCGACCACCGAGATCTACACGATCAGCATCGTCGGCAGCGTCAGATGTGTATAAGAGACAG	GATCAGCA
P5_0025A	AATGATACGGCGACCACCGAGATCTACACACACTCAGTCGTCGGCAGCGTCAGATGTGTATAAGAGACAG	ACACTCAG
P5_0026A	AATGATACGGCGACCACCGAGATCTACACTCACTATATCGTCGGCAGCGTCAGATGTGTATAAGAGACAG	TCACTATA
P5_0029A	AATGATACGGCGACCACCGAGATCTACACGCGACATATCGTCGGCAGCGTCAGATGTGTATAAGAGACAG	GCGACATA
P5_0030A	AATGATACGGCGACCACCGAGATCTACACCTATGTAGTCGTCGGCAGCGTCAGATGTGTATAAGAGACAG	CTATGTAG
P5_0031A	AATGATACGGCGACCACCGAGATCTACACCTGCTCGTTCGTCGGCAGCGTCAGATGTGTATAAGAGACAG	CTGCTCGT
P5_0032A	AATGATACGGCGACCACCGAGATCTACACGTAGTGCTTCGTCGGCAGCGTCAGATGTGTATAAGAGACAG	GTAGTGCT
P5_0033A	AATGATACGGCGACCACCGAGATCTACACATGATAGTTCGTCGGCAGCGTCAGATGTGTATAAGAGACAG	ATGATAGT
P5_0034A	AATGATACGGCGACCACCGAGATCTACACAGCAGACTTCGTCGGCAGCGTCAGATGTGTATAAGAGACAG	AGCAGACT
P5_0036A	AATGATACGGCGACCACCGAGATCTACACTGCAGCTATCGTCGGCAGCGTCAGATGTGTATAAGAGACAG	TGCAGCTA
P5_0037A	AATGATACGGCGACCACCGAGATCTACACGACAGCGTTCGTCGGCAGCGTCAGATGTGTATAAGAGACAG	GACAGCGT
P5_0039A	AATGATACGGCGACCACCGAGATCTACACCGCAGTGATCGTCGGCAGCGTCAGATGTGTATAAGAGACAG	CGCAGTGA
P5_0040A	AATGATACGGCGACCACCGAGATCTACACTCAGATGTTCGTCGGCAGCGTCAGATGTGTATAAGAGACAG	TCAGATGT
P5_0042A	AATGATACGGCGACCACCGAGATCTACACCGAGACGATCGTCGGCAGCGTCAGATGTGTATAAGAGACAG	CGAGACGA
P5_0049A	AATGATACGGCGACCACCGAGATCTACACGTGAGTGTTCGTCGGCAGCGTCAGATGTGTATAAGAGACAG	GTGAGTGT
P5_0050A	AATGATACGGCGACCACCGAGATCTACACGATGCACTTCGTCGGCAGCGTCAGATGTGTATAAGAGACAG	GATGCACT

P7 Index Name	Index Primer Sequence (5'-3')	ndex Sequence
P7_0001	CAAGCAGAAGACGGCATACGAGATTCGTCAGAGTCTCGTGGGCTCGGAGATGTGTATAAGAGACAG	TCTGACGA
P7_0002	CAAGCAGAAGACGGCATACGAGATCGCTAGCGGTCTCGTGGGCTCGGAGATGTGTATAAGAGACAG	CGCTAGCG
P7_0003	CAAGCAGAAGACGGCATACGAGATTCTCAGTAGTCTCGTGGGCTCGGAGATGTGTATAAGAGACAG	TACTGAGA
P7_0005	CAAGCAGAAGACGGCATACGAGATACGAGCAGGTCTCGTGGGCTCGGAGATGTGTATAAGAGACAG	CTGCTCGT
P7_0006	CAAGCAGAAGACGGCATACGAGATATACGTGAGTCTCGTGGGCTCGGAGATGTGTATAAGAGACAG	TCACGTAT
P7_0007	CAAGCAGAAGACGGCATACGAGATGTCTAGAGGTCTCGTGGGCTCGGAGATGTGTATAAGAGACAG	CTCTAGAC
P7_0009	CAAGCAGAAGACGGCATACGAGATCTCACAGAGTCTCGTGGGCTCGGAGATGTGTATAAGAGACAG	TCTGTGAG
P7_0010	CAAGCAGAAGACGGCATACGAGATAGACATAGGTCTCGTGGGCTCGGAGATGTGTATAAGAGACAG	CTATGTCT
P7_0012	CAAGCAGAAGACGGCATACGAGATTGATAGAGGTCTCGTGGGCTCGGAGATGTGTATAAGAGACAG	CTCTATCA
P7_0013	CAAGCAGAAGACGGCATACGAGATGACGACTGGTCTCGTGGGCTCGGAGATGTGTATAAGAGACAG	CAGTCGTC
P7_0014	CAAGCAGAAGACGGCATACGAGATTGTGCTCAGTCTCGTGGGCTCGGAGATGTGTATAAGAGACAG	TGAGCACA
P7_0018	CAAGCAGAAGACGGCATACGAGATTCTGCAGAGTCTCGTGGGCTCGGAGATGTGTATAAGAGACAG	TCTGCAGA
P7_0002A	CAAGCAGAAGACGGCATACGAGATATGATATCGTCTCGTGGGCTCGGAGATGTGTATAAGAGACAG	GATATCAT
P7_0003A	CAAGCAGAAGACGGCATACGAGATGTATCTAGGTCTCGTGGGCTCGGAGATGTGTATAAGAGACAG	CTAGATAC
P7_0005A	CAAGCAGAAGACGGCATACGAGATATCTAGAGGTCTCGTGGGCTCGGAGATGTGTATAAGAGACAG	CTCTAGAT
P7_0006A	CAAGCAGAAGACGGCATACGAGATGCTCTATAGTCTCGTGGGCTCGGAGATGTGTATAAGAGACAG	TATAGAGC
P7_0007A	CAAGCAGAAGACGGCATACGAGATAGTATGTAGTCTCGTGGGCTCGGAGATGTGTATAAGAGACAG	TACATACT
P7_0008A	CAAGCAGAAGACGGCATACGAGATGACTACAGGTCTCGTGGGCTCGGAGATGTGTATAAGAGACAG	CTGTAGTC
P7_0009A	CAAGCAGAAGACGGCATACGAGATATGAGCTGGTCTCGTGGGCTCGGAGATGTGTATAAGAGACAG	CAGCTCAT
P7_0010A	CAAGCAGAAGACGGCATACGAGATAGATAGACGTCTCGTGGGCTCGGAGATGTGTATAAGAGACAG	GTCTATCT
P7_0011A	CAAGCAGAAGACGGCATACGAGATCTATACAGGTCTCGTGGGCTCGGAGATGTGTATAAGAGACAG	CTGTATAG
P7_0012A	CAAGCAGAAGACGGCATACGAGATTAGATGTAGTCTCGTGGGCTCGGAGATGTGTATAAGAGACAG	TACATCTA
P7_0013A	CAAGCAGAAGACGGCATACGAGATGAGACATAGTCTCGTGGGCTCGGAGATGTGTATAAGAGACAG	TATGTCTC
P7_0014A	CAAGCAGAAGACGGCATACGAGATAGAGTGAGGTCTCGTGGGCTCGGAGATGTGTATAAGAGACAG	CTCACTCT
P7_0016A	CAAGCAGAAGACGGCATACGAGATAGACTATAGTCTCGTGGGCTCGGAGATGTGTATAAGAGACAG	TATAGTCT
P7_0019A	CAAGCAGAAGACGGCATACGAGATAGCTGAGAGTCTCGTGGGCTCGGAGATGTGTATAAGAGACAG	TCTCAGCT
P7_0022A	CAAGCAGAAGACGGCATACGAGATATGTATGAGTCTCGTGGGCTCGGAGATGTGTATAAGAGACAG	TCATACAT
P7_0024A	CAAGCAGAAGACGGCATACGAGATTCTGATAGGTCTCGTGGGCTCGGAGATGTGTATAAGAGACAG	CTATCAGA
P7_0030A	CAAGCAGAAGACGGCATACGAGATCGTATGAGGTCTCGTGGGCTCGGAGATGTGTATAAGAGACAG	CTCATACG
P7_0036A	CAAGCAGAAGACGGCATACGAGATGTCATATAGTCTCGTGGGCTCGGAGATGTGTATAAGAGACAG	TATATGAC
P7_0040A	CAAGCAGAAGACGGCATACGAGATGCAGAGAGGTCTCGTGGGCTCGGAGATGTGTATAAGAGACAG	CTCTCTGC
P7_0041A	CAAGCAGAAGACGGCATACGAGATGCATGAGAGTCTCGTGGGCTCGGAGATGTGTATAAGAGACAG	TCTCATGC
P7_0042A	CAAGCAGAAGACGGCATACGAGATGATGTGAGGTCTCGTGGGCTCGGAGATGTGTATAAGAGACAG	CTCACATC
P7_0047A	CAAGCAGAAGACGGCATACGAGATCAGTGATAGTCTCGTGGGCTCGGAGATGTGTATAAGAGACAG	TATCACTG
P7_0048A	CAAGCAGAAGACGGCATACGAGATCTCGATAGGTCTCGTGGGCTCGGAGATGTGTATAAGAGACAG	CTATCGAG
P7_0050A	CAAGCAGAAGACGGCATACGAGATGATAGTAGGTCTCGTGGGCTCGGAGATGTGTATAAGAGACAG	CTACTATC
P7_0055A	CAAGCAGAAGACGGCATACGAGATACGTACACGTCTCGTGGGCTCGGAGATGTGTATAAGAGACAG	GTGTACGT
P7_0056A	CAAGCAGAAGACGGCATACGAGATTACGTAGAGTCTCGTGGGCTCGGAGATGTGTATAAGAGACAG	TCTACGTA
P7_0068A	CAAGCAGAAGACGGCATACGAGATTGCTACGAGTCTCGTGGGCTCGGAGATGTGTATAAGAGACAG	TCGTAGCA
P7_0073A	CAAGCAGAAGACGGCATACGAGATATGACGAGGTCTCGTGGGCTCGGAGATGTGTATAAGAGACAG	CTCGTCAT
P7_0074A	CAAGCAGAAGACGGCATACGAGATTGAGACAGGTCTCGTGGGCTCGGAGATGTGTATAAGAGACAG	CTGTCTCA
P7_0078A	CAAGCAGAAGACGGCATACGAGATACTCATGAGTCTCGTGGGCTCGGAGATGTGTATAAGAGACAG	TCATGAGT
P7_0086A	CAAGCAGAAGACGGCATACGAGATACGATCGAGTCTCGTGGGCTCGGAGATGTGTATAAGAGACAG	TCGATCGT
P7_0091A	CAAGCAGAAGACGGCATACGAGATACGACGTAGTCTCGTGGGCTCGGAGATGTGTATAAGAGACAG	TACGTCGT
P7_0094A	CAAGCAGAAGACGGCATACGAGATGACGTCTAGTCTCGTGGGCTCGGAGATGTGTATAAGAGACAG	TAGACGTC
P7_0095A	CAAGCAGAAGACGGCATACGAGATCACTGTAGGTCTCGTGGGCTCGGAGATGTGTATAAGAGACAG	CTACAGTG
P7_0097A	CAAGCAGAAGACGGCATACGAGATGTATCATAGTCTCGTGGGCTCGGAGATGTGTATAAGAGACAG	TATGATAC
P7_0100A	CAAGCAGAAGACGGCATACGAGATCAGATCAGGTCTCGTGGGCTCGGAGATGTGTATAAGAGACAG	CTGATCTG
P7_0102A	CAAGCAGAAGACGGCATACGAGATAGCGACGCGTCTCGTGGGCTCGGAGATGTGTATAAGAGACAG	GCGTCGCT
P7_0103A	CAAGCAGAAGACGGCATACGAGATGCAGATCAGTCTCGTGGGCTCGGAGATGTGTATAAGAGACAG	TGATCTGC
P7_0104A	CAAGCAGAAGACGGCATACGAGATCGTAGATAGTCTCGTGGGCTCGGAGATGTGTATAAGAGACAG	TATCTACG
P7_0106A	CAAGCAGAAGACGGCATACGAGATAGTCGACAGTCTCGTGGGCTCGGAGATGTGTATAAGAGACAG	TGTCGACT
P7_0111A	CAAGCAGAAGACGGCATACGAGATGAGTCGAGGTCTCGTGGGCTCGGAGATGTGTATAAGAGACAG	CTCGACTC
P7_0112A	CAAGCAGAAGACGGCATACGAGATAGATGCAGGTCTCGTGGGCTCGGAGATGTGTATAAGAGACAG	CTGCATCT
P7_0139A	CAAGCAGAAGACGGCATACGAGATAGTGCTAGGTCTCGTGGGCTCGGAGATGTGTATAAGAGACAG	CTAGCACT

GRAS-Di markers			
	SNP markers	Amplicon markers	Total
RIL71	495	555	1050
RIL98	499	786	1285
RIL16	593	1115	1708
RIL91	635	1069	1704

Figure S2. Total number of markers generated by GRAS-Di

RIL71		GoldenGate			
		A	B	-	total
GRAS-Di SNP+ Amplicon markers	A	11080	18	14	11112
	B	28	10995	46	11069
	-	8	5	606	619
	total	11116	11018	666	22121
corresponding ratio	0.997920528005				

RIL98		GoldenGate			
		A	B	-	total
GRAS-Di SNP+ Amplicon markers	A	7291	11	14	7316
	B	11	7480	3	7494
	-	22	9	423	454
	total	7324	7500	440	14793
corresponding ratio	0.998512810113				

RIL 16		GoldenGate			
		A	B	-	total
GRAS-Di SNP+ Amplicon markers	A	9150	21	41	9212
	B	15	10574	13	10602
	-	11	31	569	611
	total	9176	10626	623	19760
corresponding ratio	0.998178137652				

RIL91		GoldenGate			
		A	B	-	total
GRAS-Di SNP+ Amplicon markers	A	10397	13	32	10442
	B	11	11214	18	11243
	-	6	8	1613	1627
	total	10414	11235	1663	21635
corresponding ratio	0.998890686388				

Figure S3. Example of calculation of correspondence ratio between makers generated by GoldenGate method and markers generated by GRAS-Di technology
